# Role of bone morphogenetic proteins in sprouting angiogenesis: differential BMP receptor-dependent signaling pathways balance stalk *vs.* tip cell competence

**DOI:** 10.1096/fj.201700193RR

**Published:** 2017-07-21

**Authors:** Andreas Benn, Christian Hiepen, Marc Osterland, Christof Schütte, An Zwijsen, Petra Knaus

**Affiliations:** * Institute for Chemistry and Biochemistry, Freie Universität Berlin, Berlin, Germany;; † Deutsche Forschungsgemeinschaft (DFG) Graduate School 1093, Berlin School of Integrative Oncology, Berlin, Germany;; ‡ DFG Graduate School 203, Berlin-Brandenburg School for Regenerative Therapies, Berlin, Germany;; § Vlaams Instituut voor Biotechnologie (VIB) Center for Brain and Disease Research, KU Leuven, Leuven, Belgium;; ¶ Department of Human Genetics, Katholieke Universiteit (KU) Leuven, Leuven, Belgium;; ‖ Zuse Institute Berlin, Berlin, Germany;; # Institute for Mathematics, Freie Universität Berlin, Berlin, Germany

**Keywords:** ALK2, ALK3, SMAD1/5, p38 MAPK, cell migration

## Abstract

Before the onset of sprouting angiogenesis, the endothelium is prepatterned for the positioning of tip and stalk cells. Both cell identities are not static, as endothelial cells (ECs) constantly compete for the tip cell position in a dynamic fashion. Here, we show that both bone morphogenetic protein 2 (BMP2) and BMP6 are proangiogenic *in vitro* and *ex vivo* and that the BMP type I receptors, activin receptor-like kinase 3 (ALK3) and ALK2, play crucial and distinct roles in this process. BMP2 activates the expression of tip cell–associated genes, such as delta-like ligand 4 (*DLL4*) and kinase insert domain receptor (*KDR*), and p38-heat shock protein 27 (HSP27)–dependent cell migration, thereby generating tip cell competence. Whereas BMP6 also triggers collective cell migration *via* the p38-HSP27 signaling axis, BMP6 induces in addition SMAD1/5 signaling, thereby promoting the expression of stalk cell–associated genes, such as hairy and enhancer of split 1 (*HES1*) and fms-like tyrosine kinase 1 (*FLT1*). Specifically, ALK3 is required for sprouting from HUVEC spheroids, whereas ALK2 represses sprout formation. We demonstrate that expression levels and respective complex formation of BMP type I receptors in ECs determine stalk *vs.* tip cell identity, thus contributing to endothelial plasticity during sprouting angiogenesis. As antiangiogenic monotherapies that target the VEGF or ALK1 pathways have not fulfilled efficacy objectives in clinical trials, the selective targeting of the ALK2/3 pathways may be an attractive new approach.—Benn, A., Hiepen, C., Osterland, M., Schütte, C., Zwijsen, A., Knaus, P. Role of bone morphogenetic proteins in sprouting angiogenesis: differential BMP receptor-dependent signaling pathways balance stalk *vs.* tip cell competence.

Expansion of the existing vasculature occurs *via* sprouting angiogenesis, a dynamic process that is orchestrated by several distinct signaling pathways, including VEGF and Notch pathways (1). VEGF-A signals *via* the kinase insert domain receptor (KDR; VEGF receptor 2) (2) and promotes filopodia formation, cell migration, and up-regulation of the Notch ligand, delta-like ligand 4 (DLL4) (3, 4). In turn, DLL4 activates Notch signaling in neighboring endothelial cells (ECs), thereby inducing the expression of hairy and enhancer of split 1 (HES1) transcription factors, such as HES1 and Hes-related family basic helix-loop-helix transcription factor with YRPW motif 1. As a result, KDR gene transcription is repressed, which ultimately renders the cell less responsive to VEGF-A (5, 6). Stalk cells maintain connectivity to the parental vessel and engage in sprout elongation, whereas the DLL4-enriched tip cells migrate toward the VEGF-A gradient, thereby guiding the nascent sprouts (4, 7). Stalk and tip cell identities are not static, as ECs constantly compete for the tip cell position in a dynamic fashion that resembles a tug of war (8). Alterations in VEGF receptor expression were described to mediate this dynamic competition. Elevated KDR levels were correlated with a predominant tip cell position occupation, whereas increased levels of fms-related tyrosine kinase 1 (FLT1; VEGF receptor 1) had the opposite effect (9). This mechanism and the lateral inhibition of tip cell identity *via* DLL4-Notch signaling dynamically balance stalk and tip cell competence to ensure correct sprout formation and coordinated elongation during sprouting angiogenesis (3, 10–12).

Several studies report that bone morphogenetic protein (BMP)–induced signaling dynamically regulates blood vessel formation (13). BMPs are members of the TGF-β family and signal *via* heterotetrameric receptor complexes that are composed of 2 type I, including activin receptor-like kinase 1 (ALK1), ALK2, ALK3, and ALK6, and 2 type II, including BMP receptor type II and activin receptor type IIa and type IIb, transmembrane serine/threonine kinase receptors (14, 15). Upon ligand binding, distinct modes of receptor oligomerization enable the transphosphorylation of the type I receptor, which displays a high affinity for the ligand, by the constitutive active type II receptor to activate intracellular signaling pathways (16, 17). Canonical signaling results in the phosphorylation and activation of receptor-regulated SMADs (R-SMADs, SMAD1/5/8) that form a heteromeric complex with the common mediator SMAD (co-SMAD, SMAD4), translocate to the nucleus, and function as transcriptional regulators to control the expression of BMP-responsive target genes, including members of the inhibitor of differentiation (ID) family of helix-loop-helix proteins (18, 19). Ligand binding also activates other signaling pathways that are collectively referred to as non-SMAD pathways. These promote transcriptional and nontranscriptional responses, the latter including actin reorganization and cell migration (17, 20). Among these non-SMAD pathways are the p38 (MAPK; hereafter referred to as p38) and PI3K pathways (21, 22).

In the endothelium, crosstalk between BMP-SMAD and Notch signaling is required for stalk cell specification during embryonic and postnatal retinal angiogenesis (23, 24), and BMP signaling regulates sprouting angiogenesis in the zebrafish axial vein in a VEGF-independent manner (25, 26). As a result of its causal relation in vascular disorders, including hereditary hemorrhagic telangiectasia, the BMP9/BMP10-ALK1 pathway has been of research interest in the past (13, 27). The essential function of ALK1 signaling in the endothelium has identified this pathway as an attractive candidate for therapeutic approaches (28–35). In contrast, comparably few mechanistic details are known about other BMP ligands, such as BMP2 and BMP6, which also regulate endothelial proliferation, migration, tube formation, and permeability (36–42). BMP2 and BMP6, in contrast to BMP9, signal *via* ALK2 and ALK3 receptors to induce SMAD and non-SMAD signaling (43). The precise function of these receptors and the balance of BMP-induced signaling pathways in ECs is poorly understood.

In this study, we investigate the function of BMP2 and BMP6 signaling in ECs and characterize them as potent angiogenic factors *in vitro* and *ex vivo*. We provide evidence that ALK3 is required for efficient sprout formation, whereas ALK2 represses endothelial outgrowth. Molecular investigations have demonstrated that BMP2 and BMP6 signal *via* distinct receptor complexes to differentially activate SMAD1/5 and p38 signaling cascades, thereby balancing stalk and tip cell competence and controlling EC migration *in vitro*.

## MATERIALS AND METHODS

### Chemicals and reagents

All chemicals were purchased from Sigma-Aldrich (Hamburg, Germany), unless otherwise indicated. Production of recombinant human BMP2 (44) and BMP6 was previously described (45). BMP type I receptor kinase inhibitor, K02288, was a gift of A. Bullock (University of Oxford, Oxford, United Kingdom) (46). BMP2 and BMP6 were used at 10 nM, unless otherwise indicated. Recombinant human VEGF-A_165_ (ImmunoTools, Friesoythe, Germany) was used at 2 nM, and recombinant human BMP2 produced in CHO cells (BMP2-CHO; PeproTech, Rocky Hill, NJ, USA) was used at 10 nM. K02288 was used at 1 µM. p38 kinase inhibitor, SB203580 (Sigma-Aldrich), was used at 20 µM (47).

### Cell culture and transfection

HUVECs were isolated as previously described (48, 49). Human pulmonary microvascular EC (HPMEC)-ST1.6R cells, herein referred to as HPMECs, were generated as previously described (50). HUVECs, HPMECs, and human aortic ECs (HAECs; PromoCell, Heidelberg, Germany) were cultured on gelatin-coated tissue culture ware in M199 medium (with Earle’s salts and NaHCO_3_) that was supplemented with 20% fetal calf serum (FCS; Biochrom AG, Berlin, Germany), 50 µg/ml EC growth supplement (Corning, Corning, NY, USA), 25 µg/ml heparin, 2 mM l-glutamine, 100 U/ml penicillin, and 0.1 mg/ml streptomycin at 37°C and 5% CO_2_. HUVECs were used between passages 2 and 5 from isolation. HAECs were used between passages 4 and 9 from isolation. Unless indicated otherwise, ECs were starved for 6 h before stimulation in M199 medium (with Earle’s salts and NaHCO_3_) that was supplemented with 2 mM l-glutamine, 100 U/ml penicillin, and 0.1 mg/ml streptomycin, herein referred to as endothelial basal medium (EBM).

Cells were treated with SB203580 60 min before stimulation.

For gene silencing, commercially available small interfering RNA (siRNA) against *ALK1*, *ALK2*, *ALK3*, *SMAD1*, *SMAD5*, and *HSPB1* (heat shock protein family B member 1; ON-TARGETplus SMARTpool; GE Healthcare, Waukesha, WI, USA) was transfected by using Lipofectamine2000 (Thermo Fisher Scientific, Darmstadt, Germany) according to manufacturer instructions. ON-TARGETplus nontargeting siRNA, herein referred to as si-scr (GE Healthcare), was used as control.

### Aortic ring assay

Mouse aortic ring assay was performed with 3- to 4-wk-old C57BL6 wild-type mice as previously described (51). Medium that contained the indicated growth factors was exchanged every other day, and images were taken after 6 d. Aortic rings were analyzed and scored in duplicates per experimental condition on phase-contrast images according to the apparent network formation. A vasculature index score (0, no vascular network; 10, vascular network completely covers optical field) was used to determine network formation.

All animal procedures were approved by the State Office of Health and Social Affairs (Berlin, Germany).

### Spheroid sprouting assay

HUVEC spheroids were generated as previously described (52). Spheroids were embedded in 20% growth factor–reduced Matrigel (Corning) in EBM that was supplemented with 0.5% FCS. Subsequently, spheroids were treated with indicated growth factors for 24 h at 37°C and 5% CO_2_.

For gene silencing spheroid sprouting assays, HUVECs were transfected with indicated siRNAs. Subsequently, spheroids were generated as described above, embedded in 20% growth factor–reduced Matrigel (Corning) in EBM that was supplemented with 5% FCS, and incubated for 24 h at 37°C and 5% CO_2_.

Total sprout length was measured from at least 3 spheroids per experimental condition on phase-contrast images by using ImageJ (National Institutes of Health, Bethesda, MD, USA).

Immunofluorescence staining of spheroids with Alexa Fluor 488 phalloidin (Cell Signaling Technology, Danvers, MA, USA) and DAPI (Sigma-Aldrich) was performed as previously described (53). Images were acquired with a Leica SP8 laser scanning confocal microscope and Leica LSX software package (Leica Microsystems, Wetzlar, Germany). Tip cells were defined as the most distal nucleus in a sprout emerging from a spheroid. Tip cells were quantified from at least 3 spheroids per experimental condition.

### Immunoblot analysis

Cell lysis, SDS-PAGE, and immunoblotting were performed as previously described (41). Primary Abs were rabbit anti–phospho-Smad1/5/8 (Ser463/465, Ser463/465, Ser465/467), rabbit anti–phospho-Smad1/5 (Ser463/465), rabbit anti–phospho-Hsp27, rabbit anti-DLL4, and rabbit anti-GAPDH (Cell Signaling Technology); rabbit anti–phospho-p38 (Thr180/Tyr182; Promega); rabbit anti-Id1 (Santa Cruz Biotechnology, Santa Cruz, CA, USA); and rabbit anti-BMPR1A and rabbit anti-Hes1 (Abcam, Cambridge, MA, USA).

### Immunofluorescence

Immunofluorescence staining was performed as previously described (41). Primary Abs were rabbit anti-Smad1 (D59D7) and rabbit anti-p38 MAPK (Cell Signaling Technology).

### Real-time quantitative PCR

Real-time quantitative PCR (qPCR) was performed as previously described (41). Primer sequences are listed in **Table 1**.

**TABLE 1.** Oligonucleotide primers for real-time qPCR

Primer	Sequence, 5'-3'
hALK1	ACAACATCCTAGGCTTCATCGC
	GGTTTGCCCTGTGTACCG
hALK2	AAGCCTGGAGCATTGGTAA
	TCACTGGGGTACTCGGAGA
hALK3	CATCTTGGAGGAGTCGTAAGAA
	TTCTGTCCTTGAACACGAGAAA
hALK6	CTGCCATAAGTGAGAAGCAAAC
	ACAACGCAAGACCTTTGGAC
hBRII	CATGGAGATGCGTAGCTGTC
	GGTTCTGAGGAAGTGCGAGT
hActRIIa	CCTGACAGCTTGCATTGCTGACTT
	TCTGCGTCGTGATCCCAACATTCT
hActRIIb	TGAAGCACGAGAACCTGCTACAGT
	GGCATACATGTCAATGCGCAGGAA
hID1	GCTGCTCTACGACATGAACG
	CCAACTGAAGGTCCCTGATG
hID2	GTGGCTGAATAAGCGGTGTT
	TGTCCTCCTTGTGAAATGGTT
hHES1	ACGACACCGGATAAACCAAA
	CCGCGAGCTATCTTTCTTCA
hFLT1	GTTCAAGGAACCTCGGACAA
	GCTCACACTGCTCATCCAAA
hKDR	AGCGATGGCCTCTTCTGTAA
	ACACGACTCCATGTTGGTCA
hDLL4	GACCACTTCGGCCACTATGT
	TTGCTGCAGTAGCCATTCTG
hGAPDH	GAAGGTGAAGGTCGGAGTC
	GAAGATGGTGATGGGATTTC

### EC migration assays

For HUVEC sheet migration, 40,000 cells/well were seeded in culture inserts (ibidi GmbH, Planegg, Germany) 24 h before the experiment. Cells were starved in basal medium that was supplemented with 0.5% FCS and 2 µg/ml mitomycin C (Sigma-Aldrich) and subsequently stimulated with indicated growth factors for 16 h. Whenever indicated, HUVECs were treated with 20 µM SB203580, 1 µM K02288, or 2 µg/ml human VEGF Ab (R&D Systems, Wiesbaden, Germany) 60 min before stimulation. Images were taken at the start and end of each experiment, and gap closure was determined on phase-contrast images by using the Time Lapse Analyzer wound-healing software package (54).

For transwell migration, HUVECs were starved in EBM that was supplemented with 0.5% FCS for 18 h at 37°C and 5% CO^2^. Subsequently, 50,000 cells/well were seeded in the upper chamber of gelatin-coated transwell inserts (8.0-µm pore size; Corning) in EBM that was supplemented with 0.5% FCS and incubated for 2 h. Thereafter, EBM that was supplemented with 0.5% FCS and indicated growth factors were added to the lower chamber, and transwell migration was allowed for 6 h. Cells at the topside were carefully removed and cells at the lower surface were fixed with 4% paraformaldehyde, nuclei were counterstained with DAPI (Carl Roth, Karlsruhe, Germany), and filters were mounted on glass slides. Images from 5 optical fields at × 10 magnification were taken from each filter per experimental condition. Each condition was tested in duplicate. Nuclei were counted by using ImageJ.

For chemotaxis analysis, HUVECs were seeded in µ-Slide 2-dimensional chemotaxis slides (ibidi GmbH, Martinsried, Germany) according to manufacturer instructions. Cells were starved for 4 h and subsequently stimulated with indicated growth factors in a side-directed manner. Chemotaxis was observed by time-lapse microscopy for 16 h. Thirty-five to forty individual cells per experimental condition were tracked by using ImageJ and single-cell chemotaxis was analyzed by using Chemotaxis and Migration Tool software (ibidi GmbH).

Chemotaxis kinetics of HUVEC populations were analyzed by using a Markov state model and subsequent committor analysis. Image analysis was implemented in Python 2.7.10 using OpenCV 3.0 for image handling. Image segmentation was performed by using an adaptive inverse thresholding with a Gaussian window. Mitotic cells were detected by using a size-restricted circle detection. The resulting binary mask was eroded and dilated to remove artifacts from cell debris and to detach neighboring cells. Tracking was performed by using an overlap heuristic. For each object (cell) in a time step, the object of the preceding time step with the largest overlapping area was determined. During postprocessing, tracking gaps that were a result of segmentation failures in single time steps were closed by using extrapolation.

The resulting tracks were analyzed by using Markov state models. The image area was separated in a grid of 56 × 20 states and additional dummy states at the lower and upper image border, which represented the leaving of the image area. The state assignment for each cell at each time step was computed by calculating the negative exponential distance to each state, normalized to 1. To compute the transition matrix, the change in state assignment was summed up and row-normalized to 1. Subsequently, this stochastic matrix was used to calculate the committor probabilities for each state (55, 56). Here, committor probability was defined as the probability of a cell that started in a certain state to reach the lower image boundary before the upper image boundary.

### Statistical analysis

Comparison of two groups was performed by 2-tailed Student’s *t* test. Comparison of multiple groups was performed by either a 1- or 2-way ANOVA with *post hoc* Bonferroni test. Statistical calculations were performed by using SigmaPlot (Systat Software, San Jose, CA, USA) or Prism (v5; GraphPad Software, La Jolla, CA, USA). A value of *P* < 0.05 was considered statistically significant.

## RESULTS

### BMP2 and BMP6 are proangiogenic

**Figure 1.**
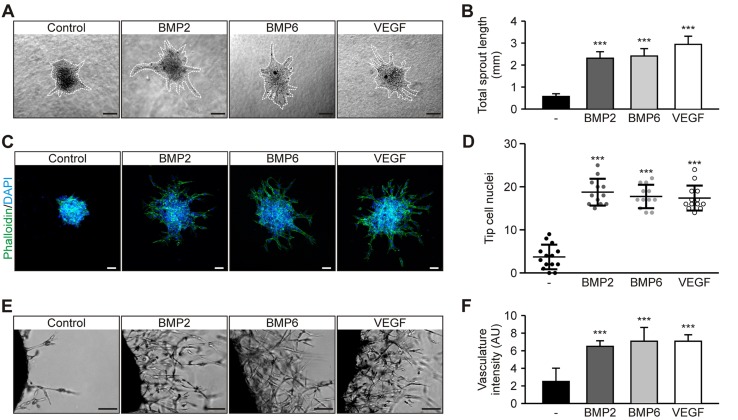
BMPs induce sprouting angiogenesis *in vitro* and *ex vivo.* *A*) Representative images of HUVEC spheroids embedded in Matrigel and treated with 10 nM BMP2, 10 nM BMP6, or 2 nM VEGF for 24 h. Dashed lines highlight spheroid sprouting area. Scale bars, 100 µm. *B*) Quantification of total sprout length. Means ± sem; *n* = 25 from 8 independent experiments. *C*) Representative confocal images of HUVEC spheroids treated with BMP2, BMP6, or VEGF and stained with phalloidin (actin; green) and DAPI (DNA; blue). Scale bars, 50 µm. *D*) Quantification of tip cell nuclei per spheroid. Means ± sd; *n* = 14 (control) and 12 (growth factors) from 3 independent experiments. *D*) Representative images of mouse aortic rings treated with BMP2, BMP6, or VEGF for 6 d. *E*) Quantification of vasculature index based on apparent capillary network formation from aortic rings. Means ± sd; *n* = 12 from 6 independent experiments. Scale bars, 100 µm. ***P < 0.001.

By using the 3-dimensional spheroid model, we demonstrate that BMP2 and BMP6 significantly increased the length of HUVEC sprouts, similar to treatment with VEGF-A_165_, hereafter referred to as VEGF, after 24 h of treatment (**Fig. 1*A*, *B***). Levels of spontaneous sprouting events in control spheroids were low (Fig. 1*A*, *B*). This correlated with a significant increase of nuclei in tip cell position (Fig. 1*C*, *D*). Furthermore, we tested the angiogenic properties of BMP2 and BMP6 by using mouse aortic rings. Aortic rings were analyzed and scored in duplicate per experimental condition on phase-contrast images according to the apparent network formation. A vasculature index score (0, no vascular network; 10, vascular network completely covers optical field) was used to determine network formation. Our results demonstrate that BMP2 and BMP6 induced capillary-like network formation from mouse aortic rings similar to VEGF (Fig. 1*E*, *F*). Taken together, we show that BMP2 and BMP6 activate ECs, thereby inducing sprout outgrowth from HUVEC spheroids and mouse aortic rings.

### BMP2 and BMP6 signal *via* distinct receptor complexes to activate SMAD1/5 and p38 pathways

**Figure 2.**
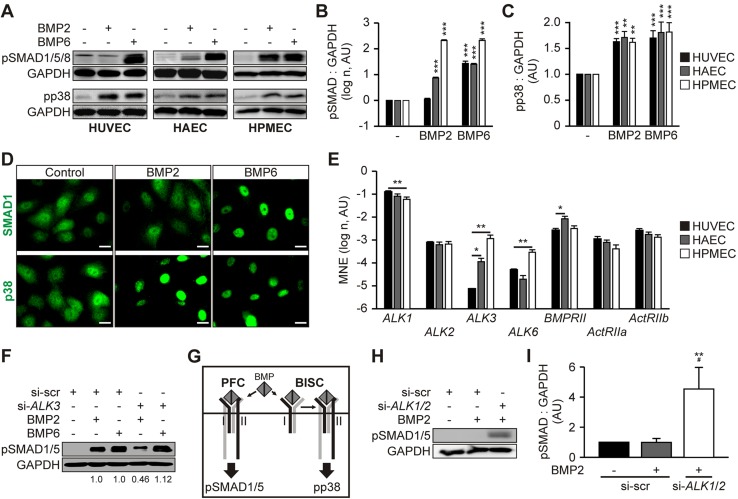
Distinct BMP type I receptor complexes determine endothelial BMP signaling. *A*) HUVECs, HAECs, and HPMECs were serum starved and subsequently stimulated with 10 nM BMP2 or BMP6 for 45 min. Cell lysates were analyzed by immunoblot with the indicated antibodies. *B*, *C*) Quantification of pSMAD1/5/8 : GAPDH (*B*) and pp38 : GAPDH (*C*) signal intensity ratio. Means ± sd; *n* = 3. *D*) Representative images of SMAD1 and p38 location in HUVECs after 30 min BMP2 or BMP6 treatment. Scale bars, 20 µm. *E*) Bar chart shows mean normalized expression (MNE) of BMP receptor transcript levels in HUVECs, HAECs, and HPMECs normalized to *GAPDH.* Means ± sem; *n* = 3. *F*) Immunoblot analysis of indicated proteins in HPMECs upon siRNA-mediated knockdown of *ALK3* and subsequent BMP2 or BMP6 stimulation for 45 min. *G*) Scheme illustrating the two modes of receptor oligomerization (preformed complex/PFC and BMP2-induced signaling complex/BISC) resulting in the induction of SMAD1/5 and p38 pathways, respectively. *H*) Immunoblot analysis of indicated proteins in HUVECs upon siRNA-mediated knockdown of *ALK1*/*2* and subsequent BMP2 stimulation for 45 min. *I*) Quantification of pSMAD1/5 : GAPDH signal intensity ratio. Means ± sd; *n* = 4. *P < 0.05, **P < 0.005, ***P < 0.001 *vs*. control or as indicated; ^#^*P* < 0.05 *vs*. BMP2-treated si-scr control.

We next addressed the underlying molecular mechanisms of BMP2- and BMP6-induced EC activation. HUVECs, HAECs, and HPMECs—covering a variety of ECs from different vascular beds—were stimulated with BMP2 or BMP6. BMP2 did not trigger SMAD1/5/8 activation in HUVECs, had an intermediate effect on HAECs, and induced SMAD1/5/8 phosphorylation similar to BMP6 in HPMECs (**Fig. 2*A*, *B***). In contrast, BMP6 promoted a comparable activation of SMAD signaling in all 3 cell types (Fig. 2*A*, *B*). The effects of BMP2 and BMP6 on HUVECs were independent of dose, time, and cell density (Supplemental Fig. 1*A*–*C*). Despite the requirement of BMP6 glycosylation for efficient ligand-receptor interaction (57), glycosylation of BMP2 did not alter its effects on canonical SMAD signaling in HUVECs or HPMECs (Supplemental Fig. 1*D*). In contrast to SMAD1/5/8 signaling, BMP2 and BMP6 promoted a likewise activation of p38 in HUVECs, HAECs, and HPMECs (Fig. 2*A*, *C*). These differential effects were also observed on nuclear translocation of SMAD1 and p38 (Fig. 2*D*). These findings indicate that activation of SMAD1/5/8 signaling is dependent on the ligand and the endothelial origin, whereas BMP-induced p38 signaling shows a cell type–independent activation for both ligands.

Subsequently, we determined the expression levels of the signal-transducing BMP receptor kinases. ALK3 mRNA and protein expression was low in HUVECs, intermediate in HAECs, and high in HPMECs (Fig. 2*E* and Supplemental Fig. 1*E*). This *ALK3* expression pattern correlated with the differential responsiveness toward BMP2 (Fig. 2*A*, *B*), which suggests that BMP2 signals *via* ALK3 to induce canonical SMAD signaling. siRNA-mediated knockdown of *ALK3* significantly reduced BMP2-induced SMAD1/5 signaling in HPMECs (Fig. 2*F*). In line with previously published results (57, 58), the pan-activity of BMP6-induced SMAD signaling (Fig. 2*A*–*C*) strongly correlated with a comparable ALK2 mRNA and protein expression in all tested ECs (Fig. 2*E* and Supplemental Fig. 1*E*). Accordingly, BMP6-induced SMAD1/5 activation was diminished by siRNA-mediated knockdown of *ALK2* in HPMECs and HUVECs (Supplemental Fig. 1*F*–*H*). This suggests that BMP6 signals *via* ALK2. Type II receptors were comparably expressed in investigated ECs (Fig. 2*E*), which indicates that the expression of distinct type I receptors confers responsiveness of endothelial SMAD1/5 signaling to different BMP ligands. Conversely, ALK3 expression did not correlate with BMP2-induced p38 signaling (Fig. 2*A*, *E*), which suggests that BMP-induced SMAD1/5 and p38 signaling pathways require different receptor oligomerization.

We have previously shown that SMAD1/5 and p38 signaling require different modes of receptor oligomerization, and have reported that binding of BMP2 to preformed complexes (PFCs) of type I and type II receptors activates SMAD1/5/8 signaling (59). BMP2 can also first bind to its high-affinity type I receptor ALK3 and subsequently recruit type II receptors into the complex. This mode of receptor oligomerization has been termed BMP2-induced signaling complex (BISC), and preferentially activates p38 (59) (Fig. 2*G*). This model implies that type I receptor expression levels determine signaling outcomes, as low levels of ALK3 still allow BISC formation, whereas ALK3 might be outcompeted for type II receptor binding by other type I receptors within PFCs. On the basis of mRNA expression data, we assumed that ALK1 and ALK2 predominantly occupy the type I receptor position in PFCs and undermine ALK3 incorporation as a result of its low abundance (Fig. 2*E*). To assess whether changes in type I receptor stoichiometry modulate BMP2-dependent signaling, we reduced the levels of ALK1 and ALK2, which theoretically allowed the incorporation of ALK3 into PFCs. Upon siRNA-mediated knockdown of *ALK1* and *ALK2*, HUVECs regained BMP2-induced SMAD1/5 activation (Fig. 2*H*, *I* and Supplemental Fig. 1*F*). Knockdown of *ALK1* and *ALK2* did not affect BMP receptor expression stoichiometry (Supplemental Fig. 1*I*), which suggests that BMP2-dependent signaling is mediated *via* distinct receptor complexes. Taken together, we demonstrate that BMP2 activates p38 signaling, whereas BMP6 promotes phosphorylation of SMAD1/5 and p38 in HUVECs. These differential signaling outcomes seem to reflect the incorporation of specific BMP type I receptors into functional receptor complexes.

### ALK2 and ALK3 have opposing roles in sprouting angiogenesis

**Figure 3.**
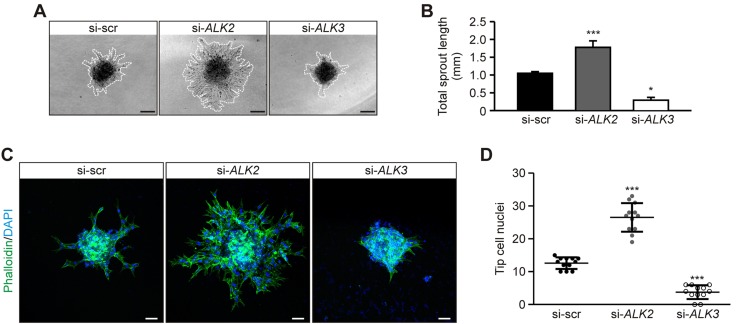
ALK2 and ALK3 have opposing roles during *in vitro* sprouting angiogenesis. *A*) Representative images of HUVEC spheroids transfected with siRNAs targeting *ALK2*, *ALK3*, or control sequences (scrambled; si-scr). Dashed lines highlight spheroid sprouting area. Scale bars, 100 µm. *B*) Quantification of total sprout length. Means ± sd; *n* = 12 from 4 independent experiments. *C*) Representative confocal images of HUVEC spheroids transfected with indicated siRNA and stained with phalloidin (actin; green) and DAPI (DNA; blue). Scale bars, 50 µm. *D*) Quantification of tip cell nuclei per spheroid. Means ± sd; *n* = 12 from 4 independent experiments. *P < 0.05, ***P < 0.001.

Our data suggest that BMP2 signals *via* ALK3, whereas BMP6-dependent signaling is mediated *via* ALK2. As both ligands are proangiogenic, we investigated the function of ALK2 and ALK3 during sprouting angiogenesis. HUVECs were transfected with siRNA that targeted either *ALK2* or *ALK3* and were used to generate spheroids. Outgrowth from these spheroids was subsequently analyzed. Upon *ALK2* knockdown, hypersprouting was observed compared with control transfected spheroids, whereas *ALK3* knockdown resulted in significantly less sprouting (**Fig. 3*A*, *B***).

These findings indicate that ALK3 is required for efficient sprout formation, whereas ALK2 represses sprouting. Sprout formation requires tip cell formation (4); therefore, we investigated the functional importance of ALK2 and ALK3 expression on tip cell formation. *ALK2* knockdown significantly increased the percentage of nuclei in tip cell position compared with control transfected cells, whereas *ALK3* knockdown had the opposite effect (Fig. 3*C*, *D*). By using the spheroid model, these results demonstrate that ALK2 and ALK3 have opposing roles in sprouting angiogenesis.

### BMP-dependent SMAD1/5 and p38 signaling pathways balance stalk and tip cell competence

**Figure 4.**
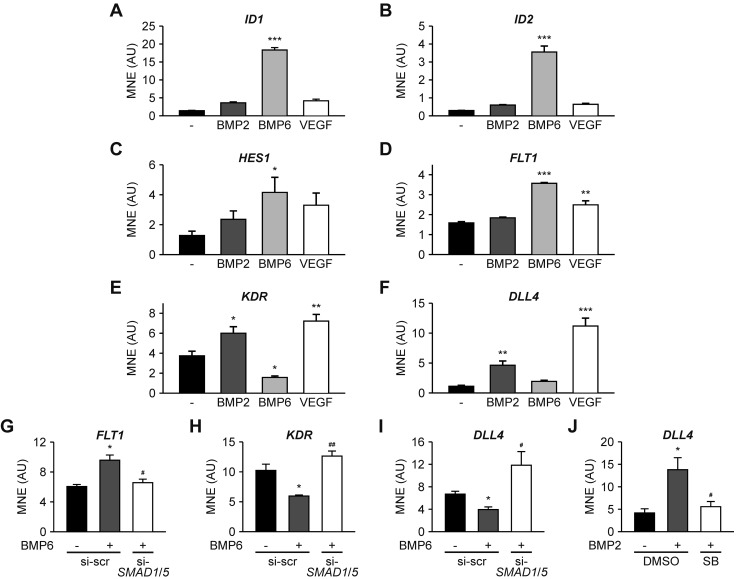
SMAD1/5 and p38 signaling differentially regulate stalk and tip cell competence. HUVECs were serum starved and subsequently treated with 10 nM BMP2, 10 nM BMP6, or 2 nM VEGF for 24 h, and levels of indicated transcripts were analyzed by real-time qPCR and normalized to *GAPDH*. *A*–*F*) *ID1* (*A*), *ID2* (*B*), *HES1* (*C*), *FLT1* (*D*), *KDR* (*E*) (*n* = 4) and *DLL4* (*F*) (*n* = 3). *G*–*I*) Transcript levels of *FLT1* (*G*), *KDR* (*H*), and *DLL4* (*I*) from si-*SMAD1*/5-transfected HUVECs upon stimulation with BMP6 for 24 h (*n* = 3). *J*) *DLL4* levels upon BMP2 stimulation in the absence or presence of 20 µM SB203580 (SB) (*n* = 3). *P < 0.05, **P < 0.005, ***P < 0.001 *vs*. control; ^#^*P* < 0.05; ^#^^#^*P* < 0.005 *vs*. BMP2-treated DMSO control.

Whereas ALK3 is required for efficient tip cell formation, ALK2 represses sprout formation, yet their respective ligands, BMP2 and BMP6, are both proangiogenic (Fig. 1*A*, *B*). Therefore, we hypothesized that downstream signaling pathways ultimately balance functional outcomes. To test this hypothesis, we used HUVECs that allow discrimination between the function of SMAD1/5 (activated by BMP6) and p38 signaling (activated by BMP2 and BMP6) and investigated whether these respective ligands regulate stalk or tip cell competence. BMP6, but not BMP2, stimulation resulted in the up-regulation of stalk cell–associated genes *ID1*, *ID2*, *HES1*, and *FLT1* (**Fig. 4*A*–*D***). These effects were also observed on ID1 and HES1 protein levels (Supplemental Fig. 2*A*, *B*). Furthermore, we observed a differential regulation of the tip cell–associated genes *KDR* and *DLL4*. Whereas BMP6 stimulation repressed *KDR* transcript levels, BMP2 and VEGF promoted up-regulation of *KDR* and *DLL4* expression (Fig. 4*E*, *F*). These effects were also observed in DLL4 protein levels (Supplemental Fig. 2*C*).

These results indicate that SMAD1/5 signaling mediates stalk cell competence, whereas active p38 signaling induces the expression of tip cell–associated genes. To validate these findings, we altered the activity of SMAD1/5 and p38 signaling pathways. Upon siRNA-mediated knockdown of *SMAD1* and *SMAD5*, BMP6-induced up-regulation of stalk cell–associated genes *FLT1* and *ID1* was abrogated (Fig. 4*G* and Supplemental Fig. 2*B*, *C*). We observed an inverse effect on BMP6-induced down-regulation of *KDR* and *DLL4*, which were up-regulated in knockdown conditions compared with control cells (Fig. 4*H*, *I*). This suggests that BMP6-induced SMAD1/5 signaling represses tip cell competence, whereas the remaining p38 signaling in SMAD1/SMAD5-knockdown conditions reverses this repression and induces the expression of tip cell–associated genes. To confirm this finding, HUVECs were stimulated with BMP2 in the absence or presence of SB203580, a selective p38 kinase inhibitor (60). BMP2-induced up-regulation of *DLL4* was abolished in the presence of SB203580 (Fig. 4*J*), which indicates that p38 signaling regulates BMP2-induced expression of tip cell–associated genes. Collectively, these results indicate that BMP-dependent p38 and SMAD1/5 signaling balance tip and stalk cell competence, respectively.

### BMP-induced collective EC migration is mediated *via* the p38-heat shock protein 27 axis

**Figure 5.**
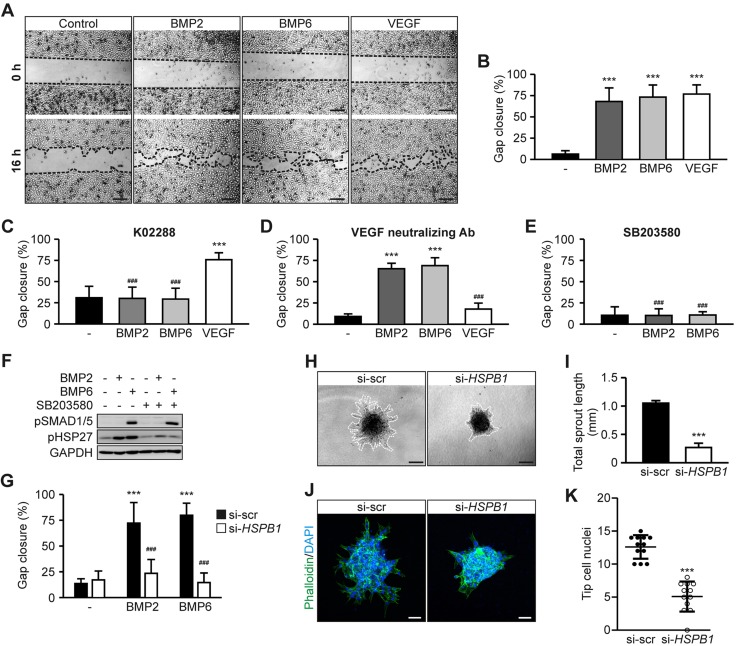
BMP-induced endothelial sheet migration is independent of VEGF and requires p38 and HSP27. *A*) Representative images of HUVEC sheets treated with 10 nM BMP2, 10 nM BMP6, or 2 nM VEGF for 16 h. Dashed lines indicate endothelial sheet borders. Scale bars, 200 µm. *B*) Quantification of HUVEC sheet migration. Means ± sd; *n* = 8. ***P < 0.001 *vs*. control. *C*) Quantification of HUVEC sheet migration in the presence of 1 µM K02288. Means ± sd; *n* = 3. ***P < 0.001 *vs*. control; ^###^*P* < 0.001 *vs*. VEGF-treated control without K02288 (as quantified in *B*). *D*) Quantification of HUVEC sheet migration in the presence of 2 µg/ml human VEGF neutralizing antibody. Means ± sd; *n* = 5. ***P < 0.001 *vs*. control; ^###^*P* < 0.001 *vs*. VEGF-treated control without anti-VEGF Ab (as quantified in *B*). *E*) Quantification of BMP-induced HUVEC sheet migration in the presence of 20 µM SB203580. Means ± sd; *n* = 8. ^###^*P* < 0.001 *vs*. BMP-treated control without SB203580 (as quantified in *B*). *F*) Immunoblot analysis of indicated proteins in HUVECs upon BMP2 or BMP6 stimulation for 45 min in the absence or presence of 20 µM SB203580. *G*) Quantification of BMP-induced sheet migration in HUVECs transfected with siRNA targeting either *HSPB1* or control sequences (scrambled; si-scr). Means ± sd; *n* = 4. ***P < 0.001 *vs*. control; ^###^*P* < 0.001 *vs*. BMP-treated si-scr control. *H*) Representative images of HUVEC spheroids transfected with siRNAs targeting *HSPB1* or control sequences (scrambled; scr). Dashed lines highlight spheroid sprouting area. Scale bars, 100 µm. *I*) Quantification of total sprout length. Means ± sd; *n* = 12 from 4 independent experiments. Control values are the same as depicted in Fig. 3*B*, as experiments were run in parallel. ***P < 0.001. *J*) Representative confocal images of HUVEC spheroids transfected with indicated siRNA and stained with phalloidin (actin; green) and DAPI (DNA; blue). Scale bars, 50 µm. *K*) Quantification of tip cell nuclei per spheroid. Means ± sd; *n* = 12 from 4 independent experiments. Control values are the same as depicted in Fig. 3*D*, as experiments were run in parallel. ***P < 0.001.

In addition to a different profile of gene transcription, migratory capacity is another determinant of tip cells in contrast to stalk cells (1, 4); therefore, we assessed whether BMP2 or BMP6 promotes collective EC migration. Upon stimulation, these endothelial sheets collectively migrate, similar to collective cell migration of a nascent sprout (61, 62). HUVEC sheet migration was performed in presence of mitomycin C to inhibit proliferation. A significant increase in HUVEC sheet migration was observed upon treatment with BMP2 and BMP6, comparable to VEGF-stimulated cells (**Fig. 5*A*, *B***). Similar results were observed with proliferation-independent transwell migration (Supplemental Fig. 3*A*). To validate the specificity of BMP2- and BMP6-induced sheet migration, we blocked BMP type I receptor kinase activity by using K02288 (46). This resulted in a complete inhibition of BMP-induced sheet migration, whereas VEGF-induced migration was unaffected (Fig. 5*C* and Supplemental Fig. 3*B*). BMPs have been shown to induce VEGF expression (63, 64), and BMP4-dependent EC migration was reported to be mediated by the activation of VEGF signaling (65); however, by using a VEGF neutralizing Ab, BMP2- and BMP6-dependent HUVEC sheet migration was still induced, whereas VEGF-induced migration was abolished (Fig. 5*D* and Supplemental Fig. 3*C*). These findings indicate that BMP2- and BMP6-induced collective EC migration requires type I receptor kinase activity, but not VEGF activity.

Considering that BMP2 induces p38, but not SMAD1/5, signaling in HUVECs (Fig. 2*A*), we hypothesized that BMP2 and BMP6 promote HUVEC migration *via* p38. To confirm this, we blocked p38 kinase activity by using SB203580, which resulted in a complete inhibition of BMP2- and BMP6-induced HUVEC sheet migration (Fig. 5*E* and Supplemental Fig. 3*D*). In addition, BMP2- and BMP6-induced sheet migration still occurred upon knockdown of *SMAD1/SMAD5* (Supplemental Fig. 3*E*, *F*), which indicates that BMP-induced migration requires p38 kinase activity independent of SMAD1/5 signaling.

As p38-dependent EC migration was demonstrated to be mediated *via* actin regulator, heat shock protein 27 (HSP27) (66), we assessed whether BMP2 or BMP6 promotes collective EC migration *via* HSP27. Both ligands induced the phosphorylation of HSP27 (Fig. 5*F*), a prerequisite for its actin stabilizing function (66). This phosphorylation was susceptible to p38 kinase inhibition (Fig. 5*F*). HUVECs that were transfected with siRNA that targeted the *HSPB1* mRNA (HSP27 gene transcript) demonstrated significantly less BMP2- and BMP6-induced sheet migration when compared with control-transfected cells (Fig. 5*G* and Supplemental Fig. 3*G*, *H*). In addition, knockdown of *HSPB1* results in significantly decreased endothelial outgrowth in the spheroid sprouting model and a reduced percentage of tip cell nuclei (Fig. 5*H*–*K*). These findings suggest that BMP2 and BMP6 specifically promote collective EC migration *via* the p38-HSP27 signaling axis and highlight HSP27 as an important regulator of endothelial outgrowth.

### BMP2 and BMP6 are potent chemoattractants

**Figure 6.**
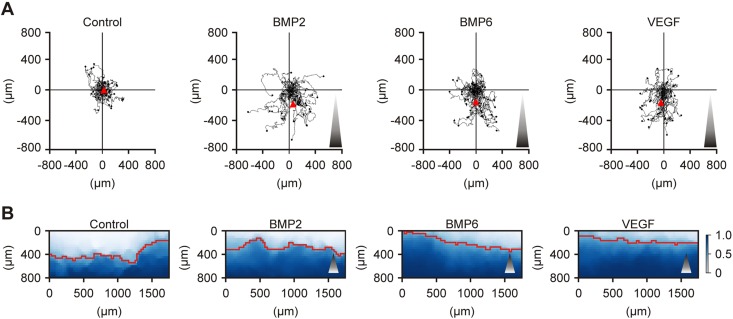
BMPs promote HUVEC chemotaxis. *A*) HUVEC single-cell chemotaxis analysis showing the tracks of 35-40 individual cells upon side-directed treatment with 10 nM BMP2, 10 nM BMP6 or 2 nM VEGF for 16 h. A representative of 3 independent experiments is shown. *B*) Markov committor analysis of collective chemotaxis upon side-directed stimulation with indicated growth factors within 4 h. All HUVECs per optical field were tracked and analyzed using Markov state models. The red isoline separates the two zones in which cells are more likely to migrate upward or downward according to committor probabilities. Lighter area: higher chance of upward migration. Darker area: higher chance of downward migration. Shaded triangles mark the direction of the growth factor gradient.

Whereas the sprout migrates collectively, tip cells constantly scan the environment, sensing gradients of chemoattractants to determine the direction of migration (4). To test whether BMP2 or BMP6 act as endothelial chemoattractants, we used a 2-dimensional assay that allows chemotaxis analysis in a gradient- and time-resolved manner (22). In the presence of a growth factor gradient, chemotaxis occurred during a 16-h observation period, whereas in the absence of a gradient, we observed only random motion (chemokinesis; **Fig. 6*A***). BMP2 and BMP6 significantly induced directed migration similar to the potent chemoattractant, VEGF (Supplemental Fig. 4*A*). The migratory capacity and velocity were only mildly affected compared with control-treated cells (Supplemental Fig. 4*B*, *C*). These findings indicate that BMP2 and BMP6 specifically promote directed EC migration.

To determine the underlying kinetics in a time-resolved manner, we developed an algorithm to track all individual cells within an optical field and performed a Markov state model and subsequent committor analysis. In the presence of a growth factor gradient, the isoline that marked the probability of chemotaxis was shifted upwards (Fig. 6*B*). This indicates that the majority of HUVECs was committed to chemotaxis already within a 4-h observation period (Fig. 6*B*). In control cells, the isoline was nearly centered; therefore, committor probabilities did not demonstrate a favored direction and correlated with chemokinesis (Fig. 6*B*). In sum, these results suggest that BMP2 and BMP6 are potent endothelial chemoattractants and rapidly initiate HUVEC chemotaxis.

## DISCUSSION

**Figure 7.**
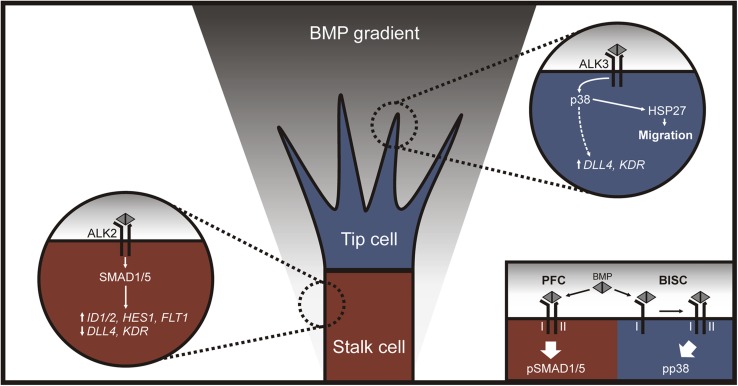
Model of an ALK2/ALK3-dependent balance of SMAD1/5 or p38 signaling in stalk and tip cell specification. Our data suggest that an ALK2-dependent activation of SMAD1/5 signaling results in expression of the stalk cell-associated genes *ID1*, *ID2*, *HES1* and *FLT1*, while the tip cell–associated genes *DLL4* and *KDR* are repressed. On the other hand, ALK3 is required for efficient sprouting and distinct receptor complexes activate p38 signaling. This results in expression of the tip cell–associated genes *DLL4* and *KDR*, as well as HSP27-dependent collective migration. Distinct BMP receptor complexes exist as preformed complexes (PFCs) or as BMP ligand-induced signaling complexes (BISCs) that activate SMAD1/5 or p38 signaling respectively. This allows a cell-autonomous balance of SMAD and non-SMAD signaling depending on expression levels and complex formation of BMP receptors.

We report that BMP2 and BMP6 are both proangiogenic *in vitro* and *ex vivo*, but their downstream signaling cascades in human ECs reveal fundamental differences. Remarkably, BMP2 activates p38 pathways without triggering canonical SMAD1/5 signaling in HUVECs. This results in the expression of tip cell–associated genes, such as *DLL4* and *KDR*, and HSP27-dependent cell migration, thereby generating tip cell competence. Whereas BMP6 also triggers collective cell migration *via* the p38-HSP27 signaling axis, BMP6-induced activation of SMAD1/5 signaling promotes the expression of stalk cell–associated genes, such as *HES1* and *FLT1*. Knockdown of SMAD1/5 demonstrates that BMP6-induced non-SMAD signaling is sufficient to induce the expression of *DLL4*, yet is repressed by otherwise active SMAD1/5 signaling. These findings may seem contradictory given that BMP-induced canonical and noncanonical signaling pathways have contrary outcomes on stalk and tip cell competence; however, we provide evidence that the activation of SMAD1/5 and p38 signaling is balanced by distinct BMP receptor complexes, thereby allowing the fine-tuning of functional outcomes in a cell-autonomous manner. Our data suggest that ALK3 forms distinct receptor complexes—the PFCs and BISCs—that activate SMAD1/5 and p38 signaling, respectively. These differential signaling outcomes were demonstrated to be mediated by a distinct lateral mobility of type I and type II receptors, which are internalized from specialized membrane microdomains *via* different endocytic routes (67–69). We show that ALK3 is required for sprouting from HUVEC spheroids, whereas ALK2 represses sprout formation; thus, we propose a model whereby ALK3-dependent activation of p38 signaling promotes tip cell competence and cell migration, whereas ALK2-dependent activation of SMAD1/5 signaling promotes stalk cell and concomitantly represses tip cell competence (**Fig. 7**).

Our model implies that expression levels and complex formation of BMP receptors determine stalk *vs.* tip cell competence, which adds another regulatory layer to endothelial plasticity during sprouting angiogenesis. It has been shown that high levels of KDR (VEGF receptor 2) are beneficial for tip cell selection, whereas ECs with high levels of FLT1 (VEGF receptor 1) are outcompeted for tip cell position (9). Of interest, we show that *FLT1* is a downstream target of SMAD1/5, whereas p38 signaling results in *KDR* expression. This suggests that BMP signaling acts upstream of VEGF receptor expression, thereby likely prepatterning the endothelium in prospective tip cells with active p38 signaling and high levels of KDR and stalk cells with active SMAD1/5 signaling and high levels of FLT1. This is in line with our observation that ID proteins demonstrate a scattered localization in the developing vasculature. SMAD1/5-dependent ID protein expression synergizes with Notch signaling to generate stalk cell competence; therefore, a scattered localization indicates that ECs with high levels of ID proteins are likely to become stalk cells, whereas low ID levels correlate with tip cell competence (23). This highlights that the endothelium is indeed prepatterned *via* a dynamic balance of signaling cascades before the onset of angiogenesis.

Recently, another study shed more light on the crosstalk between BMP-SMAD and Notch signaling in the context of lateral branching during sprouting angiogenesis (53). The authors report that Notch sets BMP responsiveness *via* Notch-dependent expression of SMAD6, an inhibitory SMAD that competes with SMAD1/5, thereby dividing stalk cells in cells with a high BMP sensitivity (low SMAD6 expression) and a low BMP sensitivity (high SMAD6 expression) (53). Stalk cells with a comparably high BMP responsiveness will most likely form lateral branches. Of interest, in addition to its inhibiting function on canonical SMAD1/5 signaling, SMAD6 has also been described to interfere with TGF-β–induced p38 activation (70). This implies that Notch-dependent SMAD6 expression might likewise interfere with canonical SMAD and non-SMAD signaling, which suggests that balancing both pathways might also be essential for functional lateral branching.

Here, we demonstrate that p38 signaling mediates tip cell competence by inducing DLL4 expression and phosphorylating HSP27. It has been demonstrated that VEGF inhibits *DLL4* repression by the Tel–C-terminal binding protein complex, which results in DLL4 expression (71). As p38 has been reported to abolish the repressor function of Tel complexes (72), this might explain the observed p38-dependent DLL4 expression in HUVECs. In addition, we characterize the p38 downstream component, HSP27, as a regulator of sprout formation in HUVEC spheroids. Of interest, whereas targeted deletion of HSP27 results in no apparent phenotype, mutant mice show defects in wound healing (73). The researchers conclude that the impaired wound healing results from defects in inflammatory reactions, yet a detailed analysis of blood vessel formation is missing (73). Considering our data and the critical role of sprouting angiogenesis during wound healing (74), this might indicate a function for HSP27 in wound healing–associated blood vessel formation. Taken together, these findings are in line with a critical function of p38-dependent signaling during blood vessel formation. Homozygous null *p38α* mutant mice die at approximately embryonic day 10.5 as a result of severe placental and embryonic vascular defects (75), which highlights that p38-dependent signaling might be an attractive pathway to further understand sprouting angiogenesis and tip cell specification.

In HUVECs, p38 also mediates BMP-induced collective cell migration independently of SMAD1/5 and VEGF activity *via* the actin capping protein, HSP27. Our findings contrast with other published studies that have reported that BMP-induced EC migration is dependent on SMAD1/5 signaling (39, 76); however, these studies either did not use HUVECs or did not address collective cell migration, which indicates that different types of migration, such as collective sheet *vs.* single-cell transwell migration, and use of ECs from different vascular beds balance SMAD and non-SMAD signaling in a different fashion. Studies that have reported that BMP2 induces transwell migration of HUVECs (38), but not of HAECs (77), support this assumption, yet detailed mechanisms remain elusive and should therefore be addressed by future studies.

Our study—in line with other recent studies—indicates a dynamic competition of stalk and tip cells that are mediated *via* different signaling modulators, such as the neuropilin 1–mediated inhibition of SMAD2/3 signaling (78) or Notch-dependent SMAD6 expression that limits SMAD1/5 signaling (53). Our model of a BMP receptor-dependent balance between SMAD1/5 and p38 signaling provides additional understanding of this dynamic process. Data from endothelial cell–specific knockout mice suggest a critical nonredundant function of ALK3 throughout the endothelium (79), whereas ALK2 has a more specialized function in the developing heart (80). Collectively, despite the prevailing research focus on BMP9-ALK1 signaling, these data highlight an equal importance of BMP2/BMP6-ALK2/ALK3 in the endothelium. This may have several clinical implications for antiangiogenic therapies during aberrant angiogenesis, such as in tumor-induced neovascularization or diabetic retinopathy. Antiangiogenic monotherapies, including targeting the VEGF pathway, resulted only in a modest normalization or failed completely (81, 82), and a promising targeting of ALK1 has recently not fulfilled the efficacy objective in phase II studies of patients with endometrial carcinoma (34) and metastatic squamous cell carcinoma (83). These drawbacks prompt the search for new antiangiogenic targets. On the basis of our findings, we propose that the balance of SMAD1/5 *vs.* p38 signaling by ALK2/ALK3 may be an attractive candidate.

## Supplementary Material

Supplemental Data

## Abbreviations

ALK: activin receptor-like kinase
BISC: bone morphogenetic protein 2–induced signaling complex
BMP: bone morphogenetic protein
DLL4: delta-like ligand 4
EBM: endothelial basal medium
EC: endothelial cell
FCS: fetal calf serum
FLT1: fms-like tyrosine kinase 1
HAEC: human aortic endothelial cell
HES1: hairy and enhancer of split 1
HPMEC: human pulmonary microvascular endothelial cell
HSP27: heat shock protein 27
HSPB1: heat shock protein family B member 1
KDR: kinase insert domain receptor
PFC: preformed complex
qPCR: quantitative PCR
siRNA: small interfering RNA
