# Supplementary material for: Role of bone morphogenetic proteins in sprouting angiogenesis: differential BMP receptor-dependent signaling pathways balance stalk vs. tip cell competence
Source: FASEB J. 2017 Jul 21;31(11):4720–33. doi: 10.1096/fj.201700193RR (PMC5636702; doi:10.1096/fj.201700193RR)
Supplement: Supplemental Data [file supp_31_11_4720__index.html]

Role of bone morphogenetic proteins in sprouting angiogenesis: differential BMP receptor-dependent signaling pathways balance stalk vs. tip cell competence — Role of bone morphogenetic proteins in sprouting angiogenesis: differential BMP receptor-dependent signaling pathways balance stalk vs. tip cell competence — Supplemental Data 

# Role of bone morphogenetic proteins in sprouting angiogenesis: differential BMP receptor-dependent signaling pathways balance stalk *vs.* tip cell competence

## Supplemental Data

- Supplemental Data
- Supplemental Data
- Supplemental Data
- Supplemental Data
