# Supplementary material for: Role of bone morphogenetic proteins in sprouting angiogenesis: differential BMP receptor-dependent signaling pathways balance stalk vs. tip cell competence
Source: FASEB J. 2017 Jul 21;31(11):4720–33. doi: 10.1096/fj.201700193RR (PMC5636702; doi:10.1096/fj.201700193RR)
Supplement: Supplemental Data [file supp_fj.201700193RR_Supplemental_Figure1.docx]

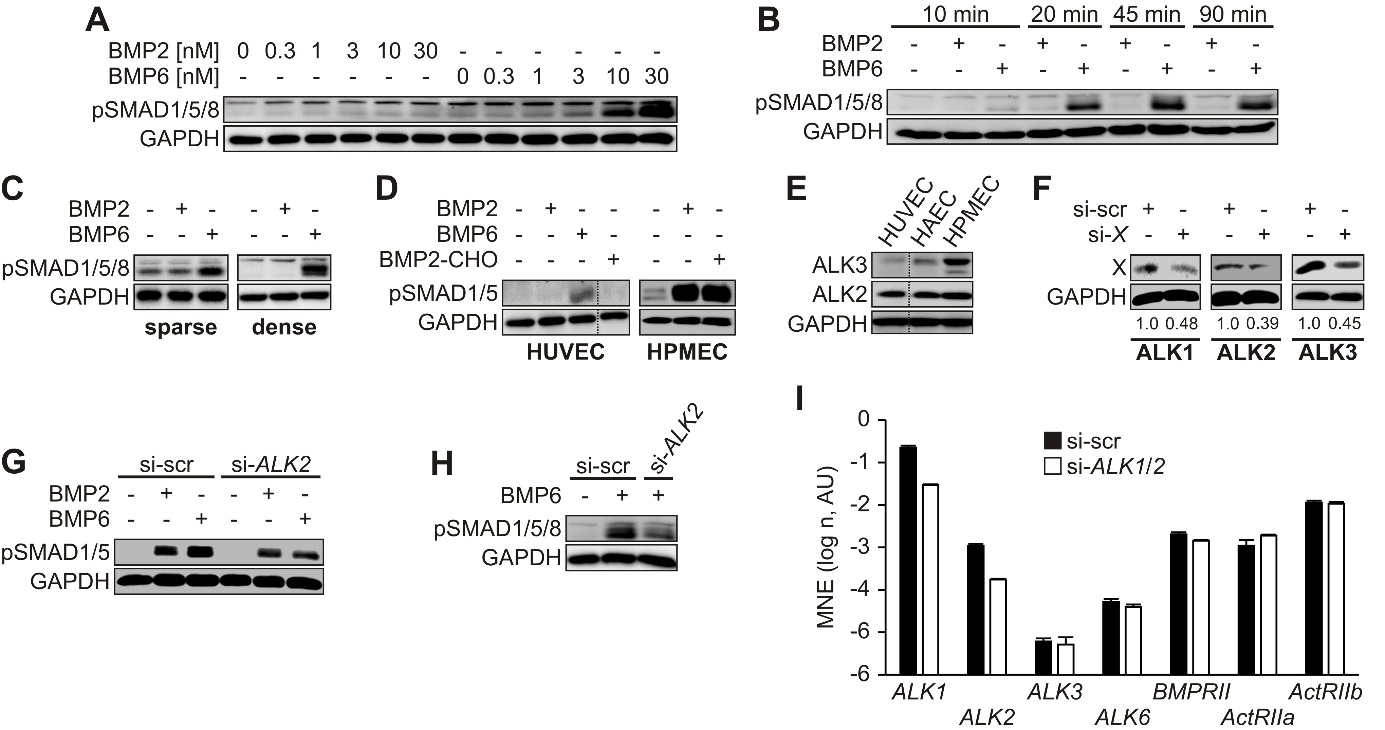


**Supplemental Figure S1 (related to Figure 2):** (A) Serum-starved HUVECs were treated with increasing concentrations of BMP2 or BMP6 for 45 min. Cell lysates were analysed by immunoblot with the indicated antibodies. (B) HUVECs were serum-starved and treated with BMP2 or BMP6 for 10, 20, 45 and 90 min. Cell lysates were analysed by immunoblot with the indicated antibodies. (C) HUVECs were seeded at a density of 15 000 cells/cm^2^ (sparse) or 100 000 cells/cm^2^ (dense). Cells were serum-starved and stimulated with BMP2 or BMP6 for 45 min. Cell lysates were analysed by immunoblot with the indicated antibodies. (D) HUVECs or HPMECs were serum-starved and treated with BMP2, BMP6 or recombinant human BMP2 produced in CHO cells (BMP2-CHO) for 45 min. Cell lysates were analysed by immunoblot with the indicated antibodies. (E) HUVECs, HAECs and HPMECs were lysed and analysed by immunoblot with the indicated antibodies. Dashed lines indicate lanes omitted from the same exposed blot membrane. (F) HUVECs were treated with siRNA against *ALK1* or *ALK2* (HUVEC) and respective protein levels were determined by immunoblot. (G) Immunoblot analysis of indicated proteins in HPMECs upon siRNA-mediated knock-down of *ALK2* and subsequent stimulation with BMP2 or BMP6 for 45 min. (H) Immunoblot analysis of indicated proteins in HUVECs upon siRNA-mediated knock-down of *ALK2* and subsequent BMP6 stimulation for 45 min. (I) Bar chart shows mean normalized expression (MNE) of BMP receptor transcript levels determined by qRT-PCR from HUVECs treated with siRNA-mediated knockdown of *ALK1/2* and normalized to *GAPDH*. Mean ± SEM.
