# Supplementary material for: Role of bone morphogenetic proteins in sprouting angiogenesis: differential BMP receptor-dependent signaling pathways balance stalk vs. tip cell competence
Source: FASEB J. 2017 Jul 21;31(11):4720–33. doi: 10.1096/fj.201700193RR (PMC5636702; doi:10.1096/fj.201700193RR)
Supplement: Supplemental Data [file supp_fj.201700193RR_Supplemental_Figure2.docx]

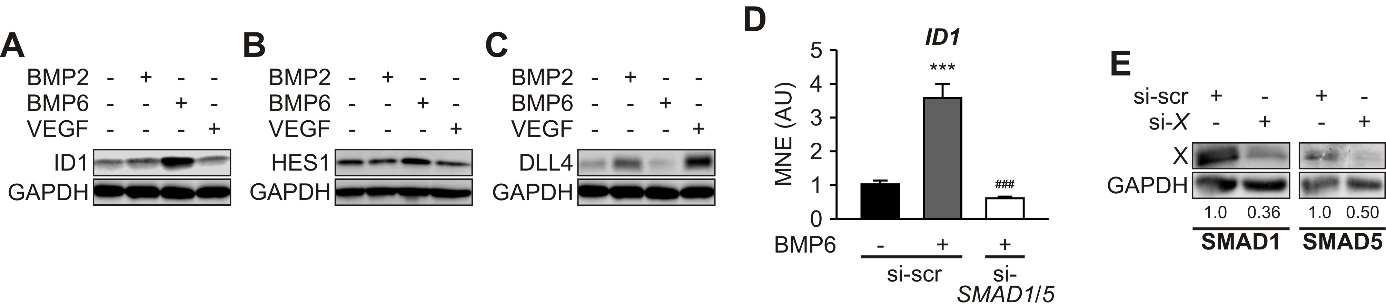


**Supplemental Figure S2 (related to Figure 4):** (A-C) HUVECs were serum-starved and stimulated with 10 nM BMP2, 10 nM BMP6 or 2 nM VEGF for 24 hours. Cell lysates were analysed by immunoblot with the indicated antibodies. (B) *ID1* transcript levels from si-*SMAD1/5* and control siRNA (scramble; si-scr) transfected HUVECs upon BMP6 stimulation for 24 hours. Mean ± SEM; n = 3. # indicates significance compared to BMP6-treated control cells, i.e. si-scr. (C) HUVECs were treated with siRNA targeting *SMAD1* or *SMAD5* and respective protein levels were determined by immunoblot.

***/### *p* < 0.001.
