# Supplementary material for: Role of bone morphogenetic proteins in sprouting angiogenesis: differential BMP receptor-dependent signaling pathways balance stalk vs. tip cell competence
Source: FASEB J. 2017 Jul 21;31(11):4720–33. doi: 10.1096/fj.201700193RR (PMC5636702; doi:10.1096/fj.201700193RR)
Supplement: Supplemental Data [file supp_fj.201700193RR_Supplemental_Figure3.docx]

**
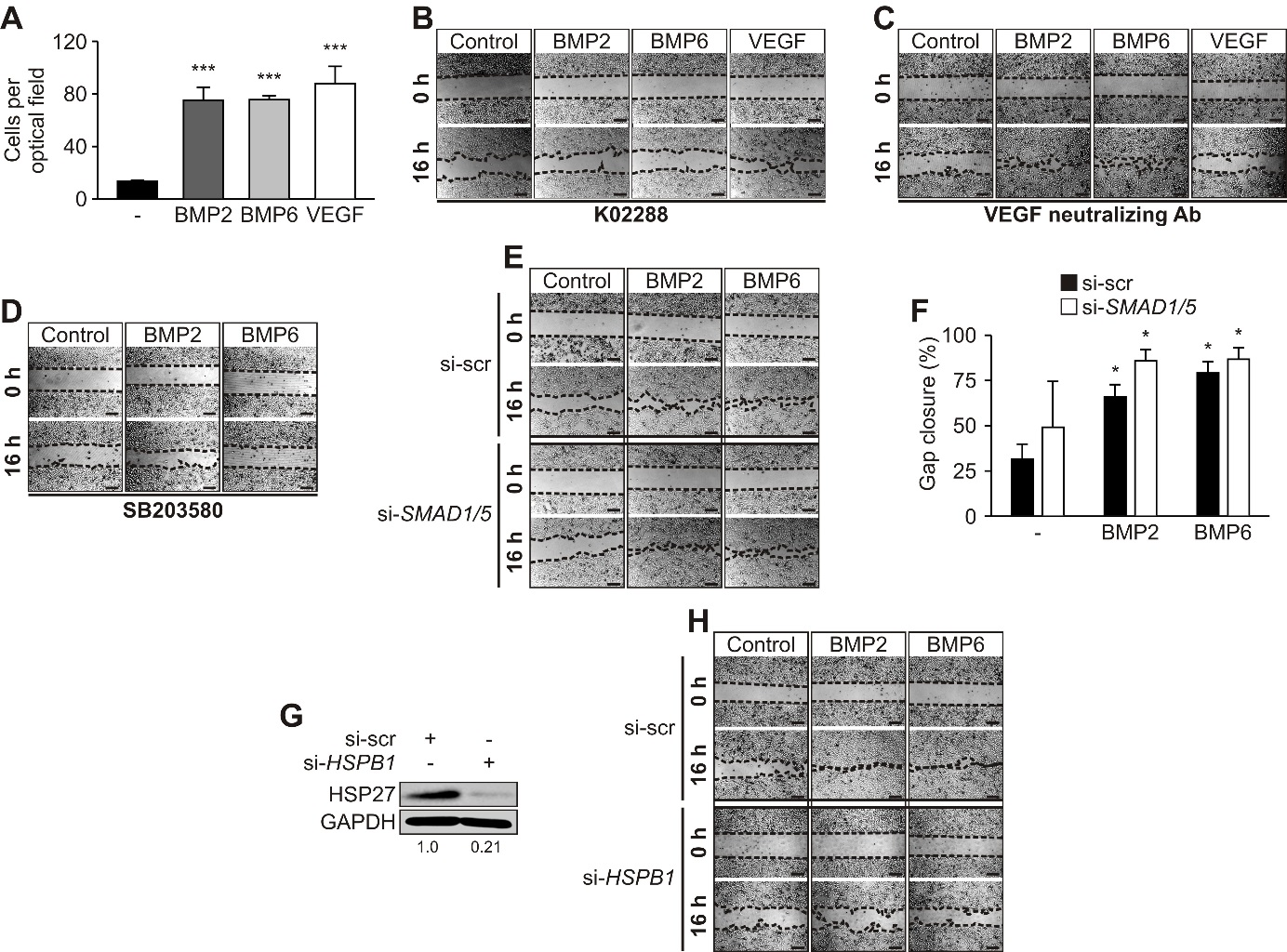
**

**Supplemental Figure S3 (related to Figure 5):** (A) HUVECs were serum-starved, seeded in transwell-inserts and stimulated with 10 nM BMP2, 10 nM BMP6 or 2 nM VEGF for 6 hours. Afterwards, migrated cells at the lower surface of the transwell-insert were fixed and nuclei were counterstained with DAPI. 5 optical fields per transwell-insert were analysed. Each condition was tested in duplicates. Mean ± SD; n = 3. (B) Representative images of HUVEC sheets treated with BMP2, BMP6 or VEGF in the presence of 1 µM K02288. (C) Representative images of HUVEC sheets treated with BMP2, BMP6 or VEGF in the presence of 2 µg/ml human VEGF neutralizing antibody. (D) Representative images of BMP-induced HUVEC sheet migration in the presence of 20 µM SB203580. (E) Representative images of BMP-induced HUVEC sheet migration upon treatment with siRNAs targeting *SMAD1/SMAD5* (HSP27) or control sequences (scrambled; si-scr). (F) Quantification of BMP-induced sheet migration in HUVECs transfected with siRNA targeting either *SMAD1/SMAD5* or control sequences (scrambled; si-scr). Mean ± SD; n = 3. (G) HUVECs were treated with siRNA targeting *HSPB1* and protein levels were determined by immunoblot. (H) Representative images of BMP-induced HUVEC sheet migration upon siRNA-mediated knockdown of *HSPB1* (HSP27) or in control cells (si-scr).

Scale bars represent 200 µm. * *p* < 0.05, *** *p* < 0.001.
