# Supplementary material for: Role of bone morphogenetic proteins in sprouting angiogenesis: differential BMP receptor-dependent signaling pathways balance stalk vs. tip cell competence
Source: FASEB J. 2017 Jul 21;31(11):4720–33. doi: 10.1096/fj.201700193RR (PMC5636702; doi:10.1096/fj.201700193RR)
Supplement: Supplemental Data [file supp_fj.201700193RR_Supplemental_Figure4.docx]

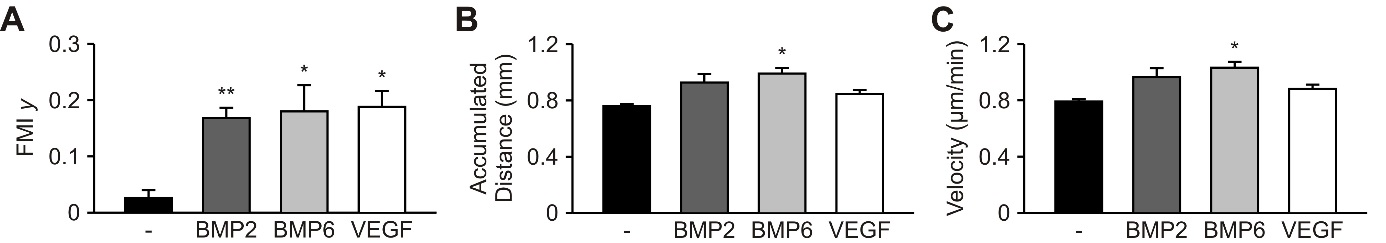


**Supplemental Figure S4 (related to Figure 6):** (A) Quantification of the mean forward migration index (FMI) y (accumulated distance/distance on y axis) of HUVEC chemotaxis upon treatment with 10 nM BMP2, 10 nM BMP6 or 2 nM VEGF for 16 hours. Mean ± SEM; n = 3. (B) Quantification of the mean accumulated distance of HUVEC chemotaxis upon treatment with BMP2, BMP6 or VEGF for 16 hours. Mean ± SEM; n = 3. (C) Quantification of mean velocity of HUVEC chemotaxis upon treatment with BMP2, BMP6 or VEGF for 16 hours. Mean ± SEM; n = 3.
